# How do common conditions impact health-related quality of life for children? Providing guidance for validating pediatric preference-based measures

**DOI:** 10.1186/s12955-023-02091-4

**Published:** 2023-01-25

**Authors:** Xiuqin Xiong, Kim Dalziel, Li Huang, Brendan Mulhern, Natalie Carvalho

**Affiliations:** 1 Centre for Health Policy, Melbourne School of Population and Global Health, The University of Melbourne, Parkville, VIC Australia; 2 Centre for Health Economics Research and Evaluation, University of Technology Sydney, Sydney, NSW Australia

**Keywords:** HRQoL, Preference-based measures, CHU9D, EQ-5D-Y, PedsQL, Validation, Dimension, Children, Known-group validity, Responsiveness

## Abstract

**Background:**

There is increasing interest in the validation of pediatric preference-based health-related quality of life measurement instruments. It is critical that children with various degrees of health-related quality of life (HRQoL) impact are included in validation studies. To inform patient sample selection for validation studies from a pragmatic perspective, this study explored HRQoL impairments between known-groups and HRQoL changes over time across 27 common chronic child health conditions and identified conditions with the largest impact on HRQoL.

**Methods:**

The health dimensions of two common preference-based HRQoL measures, the EQ-5D-Y and CHU9D, were constructed using Pediatric Quality of Life Inventory items that overlap conceptually. Data was from the Longitudinal Study of Australian Children, a nationally representative sample with over 10,000 children at baseline. Seven waves of data were included for the analysis, with child age ranging from 2 to18 years. Impacts to specific health dimensions and overall HRQoL between those having a specific condition versus not were compared using linear mixed effects models. HRQoL changes over time were obtained by calculating the HRQoL differences between two consecutive time points, grouped by “Improved” and “Worsened” health status. Comparison among various health conditions and different age groups (2–4 years, 5–12 years and 13–18 years) were made.

**Results:**

Conditions with the largest statistically significant total HRQoL impairments of having a specific condition compared with not having the condition were recurrent chest pain, autism, epilepsy, anxiety/depression, irritable bowel, recurrent back pain, recurrent abdominal pain, and attention deficit hyperactivity disorder (ADHD) for the total sample (2–18 years). Conditions with largest HRQoL improvement over time were anxiety/depression, ADHD, autism, bone/joint/muscle problem, recurrent abdominal pain, recurrent pain in other part, frequent headache, diarrhea and day-wetting. The dimensions included in EQ-5D-Y and CHU9D can generally reflect HRQoL differences and changes. The HRQoL impacts to specific health dimensions differed by condition in the expected direction. The conditions with largest HRQoL impacts differed by age group.

**Conclusions:**

The conditions with largest HRQoL impact were identified. This information is likely to be valuable for recruiting patient samples when validating pediatric preference-based HRQoL instruments pragmatically.

**Supplementary Information:**

The online version contains supplementary material available at 10.1186/s12955-023-02091-4.

## Background

Economic evaluation is increasingly used by policy makers to inform healthcare resource allocation decisions due to limited health budgets [1]. The preferred form of economic evaluation presents results as incremental cost per quality-adjusted life-years (QALYs) gained for one intervention relative to another. To calculate QALYs, preference-based health-related quality of life (HRQoL) data are required. Preference-based measures (PBMs) of HRQoL consist of two elements (descriptive systems and value sets) [2]. The focus of this paper is the validation of the descriptive system, which is important before the application of PBMs. Known-groups validity and responsiveness are the two key properties in validation studies. Known-groups validity is demonstrated when a questionnaire can discriminate between two groups known to differ on the variable of interest [3]. Responsiveness is the ability of an instrument to depict a meaningful change in an indicator between baseline and sometime later [4]. They are the properties this study trying to provide supportive evidence to.

Two popular preference-based HRQoL instruments for children and adolescents are EQ-5D-Y-3L and the Child Health Utility 9 Dimensions (CHU9D) [5]. They are the top two most applied child-specific preference-based measures [6]. EQ-5D-Y-3L is appropriate for 4–15 years old, and CHU9D is appropriate for 4–17 years old [5]. There are some studies underway trialing proxy version of CHU9D and EQ-5D-Y (both 3 level (3L) and 5L versions) with guidance notes for children 4 years old or younger [7, 8].

Pediatric Quality of Life Inventory (PedsQL) is an established, non-preference based profile generic instrument for HRQoL assessment in children and adolescents available across the age ranges of 2–18 years [9]. It is possible to use PedsQL as a proxy for the dimensions included in EQ-5D-Y and CHU9D instruments with the following reasons. PedsQL is well validated and has been used widely internationally to assess HRQoL across various heath conditions [10–12]. PedsQL has items that widely overlap with those used in pediatric PBMs [13]. Many studies have found correlations between PedsQL and EQ-5D-Y-3L and CHU9D and often use PedsQL as a gold standard for convergent validity in validation studies [4, 13–17]. There are mapping algorithms to infer EQ-5D and CHU9D utility scores from PedsQL [18–20]. Although our study focuses on providing evidence for validation for descriptive system for PBMs instead of utility (i.e., a single index which gives limited information on different health dimensions), the mapping algorithm added evidence to the common concept between PedsQL and EQ-5D-Y and CHU9D.

There are limitations with existing validation studies for EQ-5D-Y-3L and CHU9D. A frequently mentioned methodological limitation is that some dimensions of the measures are less validated [17, 21, 22]. For example, previous validation studies have found that very small proportions of children had any problems in “mobility” or “looking after myself” in the EQ-5D-Y and “daily routine” in the CHU9D, and thus had little HRQoL impairment in these dimensions [17, 21, 22]. This limits both the strength and generalizability of the validation evidence. Another issue is that there is a lack of validation studies for younger children with a range of health conditions. For example, most validation studies include children above 7 years of age for EQ-5D-Y-3L [15], and children above 11 years of age for CHU9D [13, 14, 23]. Most study samples are of school children with limited or unspecified health conditions. In addition, responsiveness is less validated [24]. There is also a lack of development of appropriate instruments for young children under 5 years [25] (i.e., PBMs for children under 5 years old are either under development or lacking validation evidence). These all contribute to the increasing interest in carrying out future validation studies [8].

It is critical that children with various degree of impairment in HRQoL or expected changes in HRQoL are included in those validation studies. Otherwise, results may be misleading and lack generalizability. Validated instruments are in turn critical for evaluation and priority setting of programs, services, treatments and supports for children and their families. Therefore, it is important for us to know more about expected HRQoL decrements and changes across a range of common childhood conditions.

The aim of this study was to investigate the HRQoL impairment and HRQoL changes over time based on dimensions captured in CHU9D and EQ-5D-Y descriptive system inferred from PedsQL across a wide range of common pediatric conditions and child age. The results can help inform the recruitment of samples in future validation studies.

## Methods

### Sample

Data were from the Longitudinal Study of Australian Children (LSAC), a geographically representative sample of Australian children and their families. The LSAC commenced in 2004, followed by repeated biennial assessment (‘waves’) of over 10,000 children across two age cohorts (a birth cohort of children aged 0–1 year and a kindergarten cohort of children aged 4–5 years in 2003–2004). The LSAC sampling design and field methods are detailed elsewhere [26]. LSAC was approved by The Australian Institute of Family Studies Ethics Committee, and families provided written informed consent. Seven waves of data (from 2004 to 2016) of both cohorts were used except for the first wave of the birth cohort because the children were under 2 years old and did not have HRQoL data collected.

### HRQoL measurement

HRQoL data were available in the form of the PedsQL (Version 4.0), which measures four health dimensions: (1) physical, (2) emotional, (3) social, and (4) school functioning and contains 23 items (21 items for 2–4 years) [27]. The PedsQL was completed by the study child’s primary caregiver, who rated the frequency of each item in the past month with a 5-point Likert scale from 0 (Never) to 4 (Almost always). Items were reversed scored and linearly transformed to a 0–100 scale (0 = 100, 1 = 75, 2 = 50, 3 = 25, 4 = 0) using recommended methods, with higher scores indicating better HRQoL [27]. In waves 6 and 7 of LSAC (children aged 10–18 years), CHU9D data (self-reported and asking about today) was also available.

### Using PedsQL as a proxy for preference based instruments

We used PedsQL items to construct scores that closely mirror the dimensions in EQ-5D-Y and CHU9D. EQ-5D-Y is the youth version of the commonly used EQ-5D, containing five dimensions: (1) mobility, (2) looking after myself, (3) usual activities, (4) pain or discomfort, and (5) worried, sad or unhappy. EQ-5D-Y has two versions, 3L (3 response levels for each dimension) and 5L (5 response levels for each dimension). The inferred EQ-5D-Y scores are based on PedsQL items and PedsQL scoring algorithm and thus do not differentiate between EQ-5D-Y-3L and EQ-5D-Y-5L. As EQ-5D-Y 3L and 5L share the same 5 dimensions, our results apply to both versions of EQ-5D-Y and thus we only use the term of “EQ-5D-Y” in the following text. CHU9D was developed for children from its inception [28], containing nine dimensions: (1) worried, (2) sad, (3) pain, (4) tired, (5) annoyed, (6) schoolwork, (7) sleep, (8) daily routine, and (9) join in activities. Each CHU9D dimension contains five levels of severity. The selection of PedsQL items to represent CHU9D dimensions was straightforward due to use of the same or very similar wording. The selection of items for EQ-5D-Y was based on the description of the dimensions and referring to Scalone et al. (2011) which proposed items expected to be correlated and correlation coefficients, supplemented with team discussion where decisions were not clear [29]. We identified PedsQL items with overlapping conceptualization to all dimensions in CHU9D and EQ-5D-Y (Additional file 1: Appendix 1). The PBMs’ dimensions that had multiple PedsQL items identified (two CHU9D dimension and three EQ-5D-Y dimensions) were checked to ensure that the items within each dimension showed significant and mostly moderate correlations (correlation interpretation criteria: weak: <0.3; moderate: 0.3-0.6; strong: >0.6) (Additional file 1: Appendix 2) [13, 30]. For each CHU9D and EQ-5D-Y dimension, all relevant PedsQL items identified were averaged to calculate a corresponding dimension score. The total score was calculated as the average of the dimension scores. Both dimension and total scores for inferred CHU9D and EQ-5D-Y range from 0 to 100, with higher scores indicating better HRQoL.

### Health conditions included

Within the LSAC survey parents reported whether their child had any ongoing health conditions, defined as a health problem that ‘exists for some period of time (weeks, months, years) or re-occurs regularly’. If the answer was ‘yes’, parents were directed to select from a group of different health conditions which varied by age. In total, 33 ongoing conditions were identified for children from 2 to 18 years old. We excluded diseases with a sample size smaller than 30 in order to keep this task manageable and focused on common health conditions as opposed to those rarely reported (leading to the exclusion of palpitation, congenital heart disease and bedwetting). We also excluded diseases that were not specific, such as “other illness” because they can provide little instructive information (leading to the exclusion of other illness, other infection, and other physical disability). There were finally 27 health conditions included (details in Additional file 1: Appendix 3).

### Statistical analyses

We described the demographic information of the study sample including gender, indigenous status, parental education, the Socio-Economic Indexes for Areas (SEIFA), and whether the child had special health care needs [30]. Children were categorized into age groups according to the age cut off points of the PedsQL: 2–4 years, 5–12 years, 13–18 years.

To estimate the HRQoL impairment of having a specific condition compared with not having that condition, we used linear mixed effects models with random intercepts accounting for the hierarchical data structure due to repeated measurement of each child in the longitudinal dataset [31]. The child identifier was used as the cluster or random intercept variable. The dependent variable is HRQoL (total or dimension scores) and the independent variable is a binary variable 1/0, where 1 indicates having a specific health condition and 0 not having the condition. The coefficient of the independent variable indicates the HRQoL impairment, i.e., HRQoL difference between groups with and without the condition. The model simply adjusted for gender and age (continuous variable in years). 15.48% of the observations had multiple conditions. Only one condition was considered for each regression model with results ranked from largest total HRQoL impairment to smallest.

To assess the responsiveness to health changes over time, the HRQoL change between two consecutive time points was calculated using the HRQoL in the current wave minus the HRQoL in the previous wave (two-year interval between waves). Three groups were defined: “Worse”, “Improved”, and “Unchanged” (detailed definition for health change in Additional file 1: Appendix 4). It is hypothesized that “Unchanged” group should have minor HRQoL changes over time, with the “Worse” group having decreased HRQoL, and the “Improved” group having increased HRQoL. The size of HRQoL change over time was assessed using the standardized response mean (SRM), which was calculated by dividing the mean change by the standard deviation of the change. The SRM can further facilitate comparison with other studies and interpretation, with SRM < 0.2 being considered small, 0.5 moderate, and 0.8 large [32].

### Sensitivity analysis

We included narrower sets of relevant item(s) for dimensions with multiple PedsQL items to see if there is any difference in the result. The detailed items included in each version of sensitivity analysis and its corresponding results are at Additional file 1: Appendix 5. We also used real CHU9D data (only available in 6th and 7th waves for 10–18 years old) to compare with the HRQoL impairment estimated using inferred CHU9D (Additional file 1: Appendix 6).

## Results

### Participants

Table 1 shows the patient characteristics of the baseline sample and the observations included in this analysis. The combined data used for the analysis were generally similar with the baseline LSAC sample. There were 52,339 observations from all eligible waves, with 8.84% of the observations missing inferred EQ-5D-Y and CHU9D measures.

**Table 1:** Patient characteristics of the study sample

Sample characteristics	Baseline sample		All the observations used (N = 9774, observations = 52,993)
	B cohort wave 2 (N = 4606)	K cohort wave 1 (N = 4983)	
Male, yes, %	51.0	51.0	51.1
Indigenous, yes, %	3.9	3.8	3.1
Special health care needs, yes, %	11.3	13.2	15.8
Primary carer's education with bachelor, yes, %	33.9	28.1	34.4
SEIFA, mean (SD)	1008.5 (74.1)	1005.7 (78.3)	1009.8 (75.5)

Descriptive information is given as arithmetic means and standard deviations (SD) or frequencies and percentages (%). The baseline sample of B Cohort for this study is wave 2 since B cohort has no HRQoL data in wave 1 (0–1 year). The sample sizes of B cohorts from Wave 2 to 7 are 4606, 4386, 4242, 4085, 3764, 3381 respectively, totaling 24,464. The sample sizes of K cohorts from Wave 1 to 7 are 4983, 4464, 4331, 4169, 3956, 3537, 3089 respectively, totaling 28,529

*SEIFA* socio-economic index for areas, *SD* standard deviation

### HRQoL impairment: regression results

**Fig. 1:**
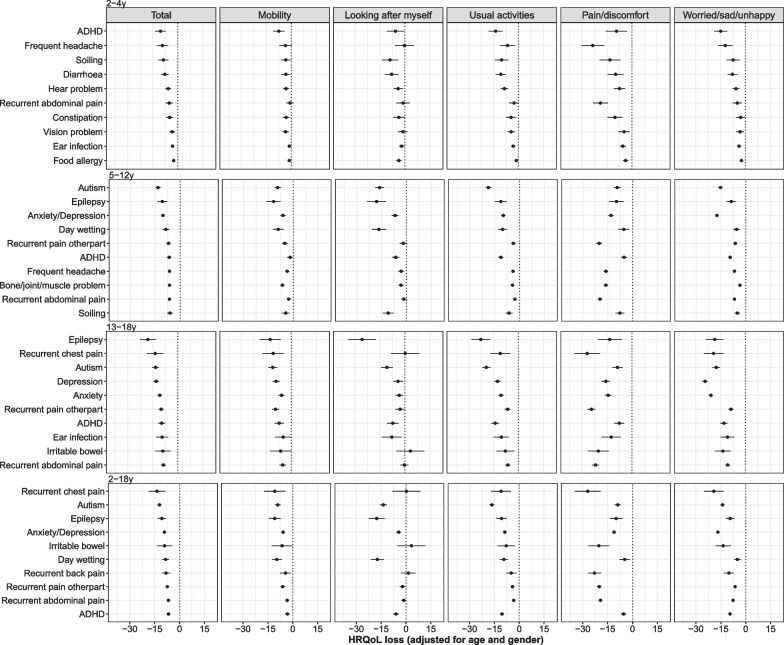
Associations between different health conditions and HRQoL across age groups based on inferred EQ-5D-Y. *Notes*: **1**. The x axis represents the HRQoL difference between those with the condition compared with those without the condition. The points are the coefficients, with the line indicating the 95% confidence interval. The conditions are ranked according to the size of the HRQoL difference. **2.** Anxiety and depression were only measured separately from the 6th wave of LSAC (10–13 years old for B cohort and 14–17 years old for K cohort), and were measured together between 4th and 7th waves (6–17 years). Thus, anxiety or depression were presented as one category in 5–12 years old age group, and as two separate categories in 13–18 years old

**Fig. 2:**
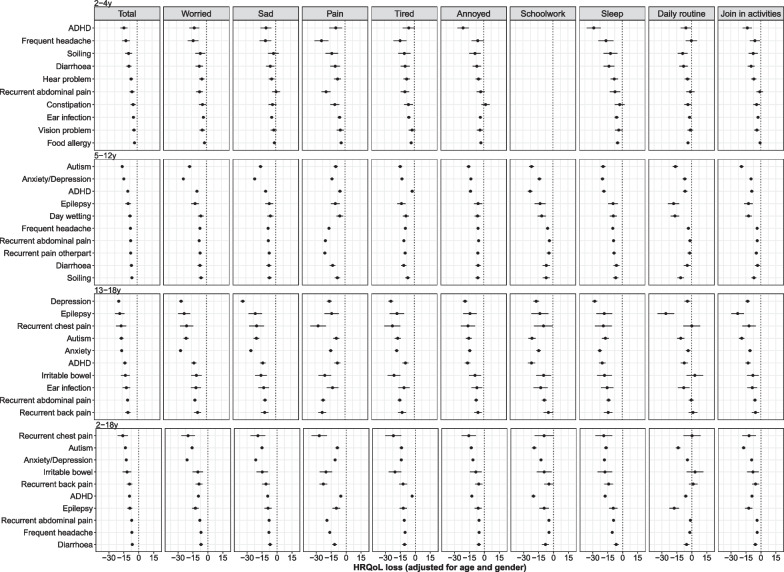
Associations between different health conditions and HRQoL across age groups based on inferred CHU9D. *Notes*:** 1.** 2–4 years old usually don’t attend school so the ‘schoolwork’ dimension is missing. **2.** Anxiety and depression were only measured separately from the 6th wave of LSAC (10–13 years old for B cohort and 14–17 years old for K cohort), and were measured together between 4th and 7th waves (6–17 years). Thus, anxiety or depression were presented as one category in 5–12 years old age group, and as two separate categories in 13–18 years old

The top 10 conditions with significant coefficients in HRQoL total scores are presented. Across all age groups, having a health condition was negatively associated with HRQoL. The shared eight conditions among the top 10 for inferred EQ-5D-Y and CHU9D are recurrent chest pain, autism, epilepsy, anxiety/depression, irritable bowel, recurrent back pain, recurrent abdominal pain, and attention deficit hyperactivity disorder (ADHD) for 2–18 year olds (Figs. 1 and 2).

The top 10 conditions were different across age groups, with some common conditions (e.g. ADHD, recurrent abdominal pain). For children aged 2–4 years the top conditions with highest HRQoL impairment include ADHD, frequent headaches, soiling and diarrhoea. For children aged 5–18 years, anxiety/depression, autism, recurrent pain and epilepsy were amongst conditions with relatively high HRQoL impairment.

In general, ‘mobility’ in EQ-5D-Y and ‘daily routine’ in CHU9D had relatively small HRQoL impairment associated with having health conditions. The influence of a health condition on various health dimensions were as expected for typical conditions. For example, for anxiety/depression, the dimensions ‘worried, sad or unhappy’, ‘worried’ and ‘sad’ were associated with the largest HRQoL impairment, while for frequent headache, recurrent pain and bone/joint/muscle problem, the dimensions ‘pain or discomfort’ and ‘pain’ showed the largest HRQoL decrement. To be noted, anxiety/depression also showed significant HRQoL impairment in the dimensions ‘pain or discomfort’ and ‘pain’.

### HRQoL change over time

**Fig. 3:**
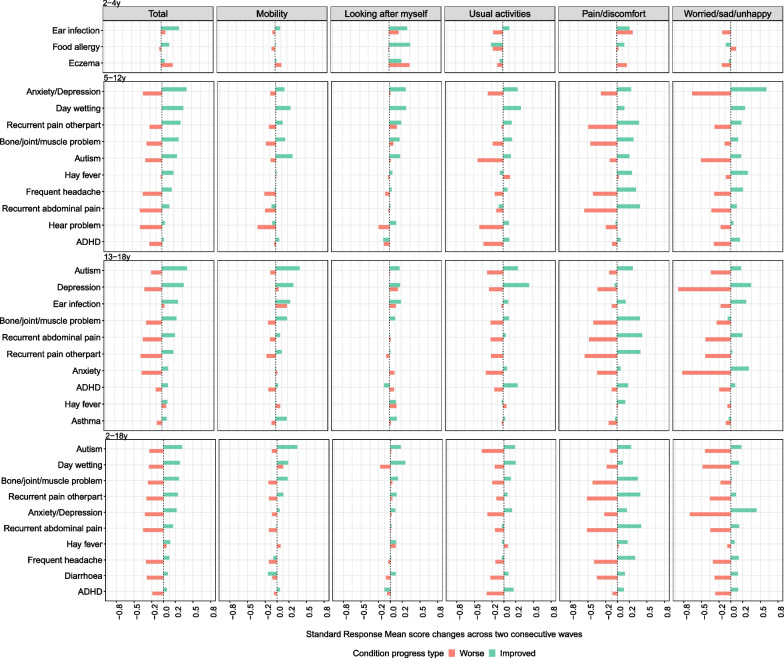
The HRQoL changes of different health conditions over a two-year period based on inferred EQ-5D-Y. *Notes*: **1.** The x axis represents the HRQoL changes measured in standard response mean (SRM). The “Improved” represents those who recovered from the condition while the “Worse” represents those who newly developed the condition. Health conditions are ranked according to the HRQoL changes of the “Improved” group. **2.** Anxiety and depression were only measured separately from the 6th wave of LSAC (10–13 years old for B cohort and 14–17 years old for K cohort), and they were measured together between 4th and 7th waves (6–17 years). Thus, anxiety or depression were presented as one category in 5–12 years old age group, and as two separate categories in 13–18 years old. **3.** Children aged 2–4 years old had only three conditions available with status changes given no HRQoL data was available for 0–1 year olds

**Fig. 4:**
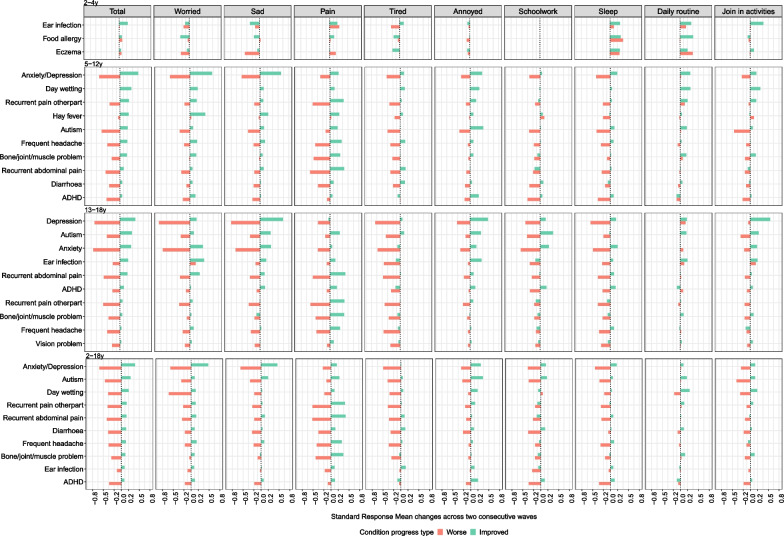
The HRQoL changes of different health conditions over a two-year period based on inferred CHU9D. *Notes*: **1.** The x axis represents the HRQoL changes measured in standard response mean (SRM). The “Improved” represents those who recovered from the condition while the “Worse” represents those who newly developed the condition. Health conditions are ranked according to the HRQoL changes of the “Improved” group. **2.** Anxiety and depression were only measured separately from the 6th wave of LSAC (10–13 years old for B cohort and 14–17 years old for K cohort), and they were measured together between 4th and 7th waves (6–17 years). Thus, anxiety or depression were presented as one category in 5–12 years old age group, and as two separate categories in 13–18 years old. **3**. Children aged 2–4 years old had only three conditions available with status changes given no HRQoL data was available for 0–1 year olds

As hypothesized HRQoL changes were generally positive in the “Improved” group and negative in the “Worse” group, except among the 2–4 year olds (Figs. 3 and 4). The HRQoL change over time was trivial among children with health condition status unchanged (Additional file 1: Appendix 4). HRQoL changes were generally larger when conditions worsened compared to when conditions improved, particularly for the inferred CHU9D. The shared nine conditions for inferred EQ-5D-Y and CHU9D among the top 10 largest SRM in the “Improved” group were anxiety/depression, ADHD, autism, bone/joint/muscle problem, recurrent abdominal pain, recurrent pain in other part, frequent headache, diarrhea and day-wetting in 2–18 years.

Around half of the top 10 conditions in all age groups except 2–4 years had small to moderate changes in overall HRQoL improvement (SRM: 0.2–0.5) for both instruments, with another half having small changes (SRM < 0.2). The 2–4 years group all had small changes (SRM < 0.2) except ear infection. Inferred CHU9D had larger HRQoL changes than inferred EQ-5D-Y, with anxiety/depression demonstrating moderate to large effects sizes (SRM: 0.5–0.8) when conditions worsened in 5–18 years old.

In 2–4 year olds, both “Improved” and “Worse” groups showed HRQoL improvement in the ‘looking after myself’, ‘pain/discomfort’ (EQ-5D-Y), ‘sleep’, ‘daily routine’ and ‘pain’ (CHU9D) dimensions. Additionally, the HRQoL change over time could be different by age group for the same condition. For example, 5–12 years old had larger HRQoL loss in ‘sleep’ and ‘annoyed’ when developing autism than 13–18 years old.

Generally, ‘pain/discomfort’ and ‘worried/sad/unhappy’ in EQ-5D-Y, and ‘worried’, ‘sad’ and ‘pain’ in CHU9D had relatively large HRQoL change over time. Again, the HRQoL changes across dimensions for typical health conditions were as expected. It is worth noting that depression had a larger HRQoL improvement than anxiety when conditions improved in 13–18 years old, with ‘mobility’ (EQ-5D-Y) and ‘join in activities’ (CHU9D) contributing most to this HRQoL increase.

### Sensitivity analyses

The analyses using narrower sets of PedsQL items to represent the EQ-5D-Y and CHU9D dimensions were consistent with the main result (Additional file 1: Appendix 5), confirming that using slightly different description and number of items had little impact on the main results. The inferred CHU9D and real CHU9D shared 5 common conditions (depression, recurrent chest pain, autism, anxiety, and soiling) among the top 10 conditions with significant HRQoL impairment. The real CHU9D data showed relatively large HRQoL impairment in recurrent conditions with consistent symptoms such as recurrent pain (back/abdominal/other part) and frequent headache. However, the inferred CHU9D demonstrated larger impact for conditions with less frequency but more severe outcomes, such as epilepsy, or with long term mental impact such as ADHD. To compare in the same condition, for example depression, real CHU9D had similar overall HRQoL impairment with inferred CHU9D. However, real CHU9D had much less impairment in ‘worried’, ‘sad’ and ‘sleep’ that are easily influenced by the mood of that particular day, but had larger impairment in ‘daily routine’ which is more stable and also with more detailed description than the inferred CHU9D (Additional file 1: Appendix 6).

## Discussion

The shared conditions among the top 10 with significant coefficients on inferred EQ-5D-Y and CHU9D total scores for 2–18 year olds were recurrent chest pain, ADHD, recurrent abdominal pain, recurrent back pain, epilepsy, anxiety/depression, irritable bowel, and autism. The shared conditions among the top 10 with largest changes in inferred EQ-5D-Y and CHU9D total score over time for 2–18 years were anxiety/depression, ADHD, autism, bone/joint/muscle problem, recurrent abdominal pain, recurrent pain in other part, frequent headache, diarrhea and day-wetting. The impacts to specific health dimensions differed by health conditions in the expected direction.

Identification of conditions with the largest HRQoL impact and which dimensions contribute to the impact may help inform the recruitment of patients in validation studies, especially for studies with limited budget and not being able to include a wide range of conditions. It is most difficult to recruit patients with large HRQoL impairment in real life. Validation studies frequently reported limitations of samples lacking severe conditions and with high ceiling effects which limited the ability to validate the instruments and suggested further research in a range of clinical conditions [13, 15, 23, 33, 34]. Future studies could consider recruiting some pediatric patients from the top 10 conditions to guarantee the effectiveness and efficiency of validation on known-group validity and responsiveness. It can also help the recruitment for multiple-instrument comparison studies, where conditions with too small HRQoL impact may limit the comparison between instruments. For a ‘real world’ example, early findings from this work informed the study design of a large validation study for multiple pediatric PBMs comparisons [8]. The results from the 2–4 years old may be especially valuable since few validation studies have included this very young population. However, the results of 2–4 years old should be interpreted with caution as EQ-5D-Y and CHU9D themselves only have experimental versions which are under evaluation and our results are based on inferred EQ-5D-Y and CHU9D from PedsQL items.

It is worth noting that 2–4 years olds had HRQoL improvements over time observed in ‘sleep’, ‘daily routine’ and ‘looking after myself’ dimensions even in the “Worse” group. One reason for this phenomenon may be that HRQoL improvements due to natural developments with age outweighs the decrease due to newly developed conditions. This might suggest that more appropriate dimensions might be needed for this young group to effectively reflect relevant HRQoL changes. Previous studies echo this suggestion [35–37]. More studies are warranted for this very young population in PBMs development or adaptation from existing measures.

Another interesting finding is that the HRQoL changes over time were generally larger when conditions were newly developed compared to when conditions resolved. One explanation for this may be that people are more sensitive to loss than gains, known as loss aversion—that is, changes for the worse (losses) seem larger than equivalent changes for the better [38, 39]. These results support the consideration of loss aversion in economic evaluations.

The consistent results from sensitivity analysis using narrower sets of PedsQL items for EQ-5D-Y and CHU9D dimensions confirmed the robustness of our results considering slightly different wording and number of items for one dimension. The common results between inferred CHU9D and real CHU9D confirmed that they had basically the same concept, while the differences may be due to difference in the degree of detailed description, recall period and proxy report or self-report (inferred CHU9D score used PedsQL items that are parent- reported over a time period of ‘the past month’, while real CHU9D is child self-reported and asked about ‘today’). The sensitivity analysis using real CHU9D data provided some support for our conceptual mapping method using PedsQL data, but also indicated the importance to consider the influence of the other factors on HRQoL measurement. The sensitivity analysis comparing inferred CHU9D and real CHU9D provided valuable information on the impact of parent proxy versus self-completion and recall period. The inferred CHU9D generally had larger or similar overall HRQoL impact than the real CHU9D (Additional file 1: Appendix 6 Fig. 2), which may suggest that parents tend to worry more about health conditions than children themselves. The real CHU9D had much less impairment in ‘worried’, ‘sad’ and ‘sleep’ dimensions that are easily influenced by random factors of the particular day of being investigated, indicating that a short recall period may reduce the ability of instruments to detect meaningful HRQoL impact on these dimensions.

Our study has several strengths. A large sample with nationally representative children in Australia (over 10, 000 children at baseline) was used. We included 27 common chronic pediatric conditions and allowed for comparison of their impact on HRQoL based on PBM constructs within a single study, which has not been reported previously. A wide childhood ages (2–18 years) enables comparison between age groups, which is valuable. We included dimensions that mirror both EQ-5D-Y and CHU9D, providing useful comparisons across instruments. Furthermore, taking advantage of the longitudinal dataset, the exploration of the HRQoL changes over time can inform responsiveness testing across a wide range of conditions which has been relatively less studied [24].

There are also some limitations. First, we used PedsQL items to mirror EQ-5D-Y and CHU9D dimensions due to not having direct PBM measurement across ages. Although the conceptualization of EQ-5D-Y and CHU9D dimensions overlapped with PedsQL items, there exist some differences, such as the exact wording of questions, the recall period and the different number of PedsQL items informing different dimensions, that may lead to different HRQoL scores. However, PedsQL has the same number of levels for each response as the CHU9D and the increasingly used EQ-5D-Y 5L, which added to its suitability to reflect the descriptive system of CHU9D and EQ-5D-Y 5L. Sensitivity analysis including narrower set of items for EQ-5D-Y dimension showed consistent results. Comparison between inferred CHU9D and real CHU9D measurement in a small subset of children showed the potential difference for appropriate interpretation and use of our results. Second, only three conditions were included in the analysis of HRQoL changes over time in 2–4 year olds due to the lack of data available for 0–1 years and small sample sizes for some health conditions. Thus, the conditions with largest HRQoL changes over time in 2–4 years old need to be interpreted with caution. Third, for 2–4 year olds, the two year interval may be too long to capture meaningful HRQoL changes over time because this young age is associated with rapid natural development whereby dimensions such as ‘daily routine’ and ‘sleep’ have a strong natural history of improvement linked to development/growing. Further HRQoL responsiveness research is needed for this age group. Finally, our analyses have focused on the descriptive systems of two common generic preference-based measures EQ-5D-Y and CHU9D, and the results may not apply to instruments with some very different health dimensions.

In conclusion, the relationship between childhood health conditions and HRQoL varies by health dimensions and age groups. Validation studies for children should include various conditions with a range of expected HRQoL impacts where relevant and possible. When there is difficulty to include patients from some disease areas, top candidates from this study may be considered based on resources and aim of the validation study.

## Supplementary Information

**Additional file 1**. Supplementary methods, results, tables and figures.

## Data Availability

Researchers who are interested in using the data could apply at the LSAC website with study protocols (https://dataverse.ada.edu.au/dataverse/ada?q=LSAC).

## Abbreviations

QALYs: Quality-adjusted life-years
HRQoL: Health-related quality of life
PBMs: Preference-based measures
CHU9D: Child Health Utility 9 Dimensions
PedsQL: Pediatric Quality of Life Inventory
LSAC: Longitudinal Study of Australian Children
SEIFA: SEIFA: Socio-Economic Indexes for Areas
SRM: Standardized response mean
ADHD: Attention deficit hyperactivity disorder

## References

[1] Krahn M (2019). Embracing the science of value in health. Can Med Assoc J.

[2] Brazier J, Deverill M (1999). A checklist for judging preference-based measures of health related quality of life: learning from psychometrics. Health Econ.

[3] Davidson M, Michalos AC (2014). Known-groups validity. Encyclopedia of quality of life and well-being research.

[4] Scott D, Ferguson GD, Jelsma J (2017). The use of the EQ-5D-Y health related quality of life outcome measure in children in the Western Cape, South Africa: psychometric properties, feasibility and usefulness—a longitudinal, analytical study. Health Qual Life Outcomes.

[5] Rowen D, et al. Review of valuation methods of preference-based measures of health for economic evaluation in child and adolescent populations: where are we now and where are we going? Pharmacoeconomics, 2020. 10.1007/s40273-019-00873-7 31903522

[6] Kwon J (2019). Patterns, trends and methodological associations in the measurement and valuation of childhood health utilities. Qual Life Res.

[7] (n.d.), T.U.o.S. Measuring & Valuing Health. A brief overview of the Child Health Utility 9D (CHU9D). Available from: https://licensing.sheffield.ac.uk/product/CHU-9D.

[8] Jones R (2021). Psychometric performance of HRQoL measures: an Australian paediatric multi-instrument comparison study protocol (P-MIC). Children (Basel, Switzerland).

[9] Varni JW, Seid M, Rode CA (1999). The PedsQL: measurement model for the pediatric quality of life inventory. Med Care.

[10] Jalali-Farahani S (2018). Comparison of health-related quality of life (HRQoL) among healthy, obese and chronically ill Iranian children. BMC Public Health.

[11] Varni JW (2012). Health-related quality of life of pediatric patients with moderate to severe plaque psoriasis: comparisons to four common chronic diseases. Eur J Pediatr.

[12] Varni JW, Limbers CA, Burwinkle TM (2007). Impaired health-related quality of life in children and adolescents with chronic conditions: a comparative analysis of 10 disease clusters and 33 disease categories/severities utilizing the PedsQL 4.0 Generic Core Scales. Health Qual Life Outcomes.

[13] Petersen KD (2019). The construct validity of the Child Health Utility 9D-DK instrument. Health Qual Life Outcomes.

[14] Yang P (2018). Psychometric evaluation of the Chinese version of the Child Health Utility 9D (CHU9D-CHN): a school-based study in China. Qual Life Res.

[15] Ravens-Sieberer U (2010). Feasibility, reliability, and validity of the EQ-5D-Y: results from a multinational study. Qual Life Res.

[16] Petersen KD (2018). Measuring health-related quality of life in adolescent populations: an empirical comparison of the CHU9D and the PedsQL(TM) 4.0 short form 15. Patient.

[17] Canaway AG, Frew EJ (2013). Measuring preference-based quality of life in children aged 6–7 years: a comparison of the performance of the CHU-9D and EQ-5D-Y–the WAVES pilot study. Qual Life Res.

[18] Shafie AA (2021). Mapping PedsQL™ Generic Core Scales to EQ-5D-3L utility scores in transfusion-dependent thalassemia patients. Eur J Health Econ.

[19] Khan KA (2014). Mapping EQ-5D utility scores from the PedsQL™ generic core scales. Pharmacoeconomics.

[20] Sweeney R (2020). Mapping PedsQL(TM) scores onto CHU9D utility scores: estimation, validation and a comparison of alternative instrument versions. Qual Life Res.

[21] Åström M (2018). Population health status based on the EQ-5D-Y-3L among adolescents in Sweden: results by sociodemographic factors and self-reported comorbidity. Qual Life Res.

[22] Chen G (2015). Assessing the health-related quality of life of Australian adolescents: an empirical comparison of the child health utility 9D and EQ-5D-Y instruments. Value Health.

[23] Ratcliffe J (2012). An assessment of the construct validity of the CHU9D in the Australian adolescent general population. Qual Life Res.

[24] Rowen D (2021). A review of the psychometric performance of selected child and adolescent preference-based measures used to produce utilities for child and adolescent health. Value Health.

[25] Rowen D (2020). Review of valuation methods of preference-based measures of health for economic evaluation in child and adolescent populations: where are we now and where are we going?. Pharmacoeconomics.

[26] Soloff C, Lawrence D, Johnstone R (2005). LSAC sample design (Technical Paper No. 1).

[27] Varni JW (2003). The PedsQL™* 4.0 as a pediatric population health measure: feasibility, reliability, and validity. Ambul Pediatr.

[28] Stevens K (2009). Developing a descriptive system for a new preference-based measure of health-related quality of life for children. Qual Life Res.

[29] Scalone L (2011). Assessing quality of life in children and adolescents: development and validation of the Italian version of the EQ-5D-Y. Ital J Public Health.

[30] Bethell CD (2002). Identifying children with special health care needs: development and evaluation of a short screening instrument. Ambul Pediatr.

[31] Diggle P (2002). Analysis of longitudinal data.

[32] Mulhern B (2018). Using generic preference-based measures in mental health: psychometric validity of the EQ-5D and SF-6D. Br J Psychiatry.

[33] Mulhern B, Meadows K (2014). The construct validity and responsiveness of the EQ-5D, SF-6D and Diabetes Health Profile-18 in type 2 diabetes. Health Qual Life Outcomes.

[34] Wolf RT (2021). The longitudinal validity of proxy-reported CHU9D. Qual Life Res.

[35] Verstraete J, Ramma L, Jelsma J (2020). Validity and reliability testing of the Toddler and Infant (TANDI) Health Related Quality of Life instrument for very young children. J Patient Rep Outcomes.

[36] Verstraete J, Ramma L, Jelsma J (2020). Item generation for a proxy health related quality of life measure in very young children. Health Qual Life Outcomes.

[37] Verstraete J (2020). How does the EQ-5D-Y Proxy version 1 perform in 3, 4 and 5-year-old children?. Health Qual Life Outcomes.

[38] Lipman SA, Brouwer WBF, Attema AE (2019). A QALY loss is a QALY loss is a QALY loss: a note on independence of loss aversion from health states. Eur J Health Econ.

[39] Kahneman D, Tversky A. Choices, values, and frames. In: Handbook of the fundamentals of financial decision making: Part I. 2013, World Scientific. p. 269–278

