# Supplementary material for: How do common conditions impact health-related quality of life for children? Providing guidance for validating pediatric preference-based measures
Source: Health Qual Life Outcomes. 2023 Jan 25;21:8. doi: 10.1186/s12955-023-02091-4 (PMC9878815; doi:10.1186/s12955-023-02091-4)
Supplement: Supplementary file 1 — Additional file 1. Supplementary methods, results, tables and figures. [file 12955_2023_2091_MOESM1_ESM.docx]

**APPENDIX**

# Appendix 1 – Relevant PedsQL items identified for EQ-5D-Y and CHU9D

**Appendix 1** **Table**

| Dimension | Relevant PedsQL items identified |
| --- | --- |
| EQ-5D-Y dimensions |  |
| Mobility (Walking about) | 1.1 Walking more than 100 meters |
|  | 1.2 Running |
| Looking after myself (washing or dressing) | 1.5 Taking a bath or shower by him or herself |
| Doing usual activities (for example, going to school, hobbies, sports, playing, doing things with family or friends) | 1.3 Participating in sports activity or exercise |
|  | 1.6 Doing chores around the house |
|  | 3.4 Not being able to do things that other children his or her age can do |
|  | 3.5 Keeping up when playing with other children |
|  | 4.3 Keeping up with schoolwork |
| Having pain and discomfort | 1.7 Getting aches and pains |
| Feeling worried, sad and or unhappy | 1.8 Having a low energy |
|  | 2.1 Feeling afraid or scared |
|  | 2.2 Feeling sad |
|  | 2.3 Feeling angry |
|  | 2.4 Having trouble sleeping |
|  | 2.5 Worrying about what will happen to him or her |
| CHU9D dimensions |  |
| Worried | 2.1 Feeling afraid or scared |
|  | 2.5 Worrying about what will happen to him or her |
| Sad | 2.2 Feeling sad |
| Pain | 1.7 Getting aches and pains |
| Tired | 1.8 Having a low energy level |
| Annoyed | 2.3 Feeling angry |
| School work/ homework | 4.3 Keeping up with schoolwork |
| Sleep | 2.4 Having trouble sleeping |
| Daily routine (eating, having a bath/ Shower, getting dressed) | 1.5 Taking a bath or shower by him or herself |
| Able to join in activities (playing out with friends, doing sports, joining things) | 1.3 Participating in sports activity or exercise  3.1 Getting along with other children  3.5 Keeping up when playing with other children |

**Note:** **1.** The description of the PedsQL items used the 8-12 years parent reported version. The different versions by age are mostly the same, with a few word differences in description. **2.** The relevant PedsQL items for EQ-5D-Y dimensions are based on the description of the dimension and referred to in the literature: Scalone, L., et al. (2011). "Assessing Quality of Life in Children and Adolescents: Development and Validation of the Italian Version of EQ-5D-Y." **3.** We chose the PedsQL items that most closely matched the description of CHU9D dimensions. When it was unclear whether an item was relevant, we took a conservative approach and excluded the item.

# Appendix 2 – Correlation of identified PedsQL items within one dimension

**Appendix 2 Table**

Internal correlation of PedsQL items representing CHU9D dimension ‘worried’

|  | Feeling afraid or scared |
| --- | --- |
| Worrying about what will happen to him or her | 0.4962* |

Internal correlation of PedsQL items representing CHU9D dimension ‘Join in activities’

|  | Participating in sports activity | Getting along with other children |
| --- | --- | --- |
| Getting along with other children | 0.3851* |  |
| Keeping up when playing with other children | 0.4809* | 0.5218* |

Internal correlation of PedsQL items representing EQ-5D-Y dimension ‘mobility’

|  | Walking more than 100 meters |
| --- | --- |
| Running | 0.7448* |

Internal correlation of PedsQL items representing EQ-5D-Y dimension ‘usual activities’

|  | Participating in sports activity | Doing chores | Not being able to do things that other children can do | Keeping up when playing with other children |
| --- | --- | --- | --- | --- |
| Doing chores | 0.3356* |  |  |  |
| Not being able to do things that other children can do | 0.3407* | 0.2408* |  |  |
| Keeping up when playing with other children | 0.4809* | 0.2672* | 0.5272* |  |
| Keeping up with schoolwork | 0.3499* | 0.3180* | 0.3941* | 0.5205* |

Internal correlation of PedsQL items representing EQ-5D-Y dimension ‘worried, sad or unhappy’

|  | Having a low energy | Feeling afraid or scared | Feeling sad | Feeling angry | Having trouble sleeping |
| --- | --- | --- | --- | --- | --- |
| Feeling afraid or scared | 0.3607* |  |  |  |  |
| Feeling sad | 0.3998* | 0.5143* |  |  |  |
| Feeling angry | 0.3013* | 0.3905* | 0.5024* |  |  |
| Having trouble sleeping | 0.3384* | 0.3671* | 0.3618* | 0.3336* |  |
| Worrying about what will happen to him or her | 0.3304* | 0.4962* | 0.5110* | 0.3663* | 0.4042* |

* *p* value <0.01.

**Note:** Most researchers agree <0.1 indicates negligible correlation, >0.9 very strong correlation. Values in between are disputable. Many studies have used 0.30 as the cutoff between weak and moderate correlation. ^2-4^

# Appendix 3 – Health conditions included in the analysis

The health conditions finally included were eczema, vision problem, hay fever, asthma, acne, food allergy, ear infection, anxiety, depression, bone/joint/muscle problem, frequent headache, recurrent adnominal pain, hearing problem, tonsillitis, constipation, recurrent back pain, autism, recurrent pain in other part, attention deficit hyperactivity disorder (ADHD), soiling, day-wetting, irritable bowel, diarrhea, recurrent chest pain, epilepsy, chronic fatigue and diabetes (27 conditions in total).

# Appendix 4 – The HRQoL change over time including the unchanged group (‘00’ and ‘11’)

For every two waves, there were four possible states that a child could be in: 1) not having one health condition in the previous wave or the current wave, ‘00’; 2) not having the condition in the previous wave, but having it in the current wave, ‘01’; 3) having the condition in the previous wave, but not having it in the current wave; ‘10’; 4) having the condition in both waves, ‘11’. Three groups were defined: “Worse” (‘01’), “Improved” (‘10’), and “Unchanged” (‘00’, ‘11’).


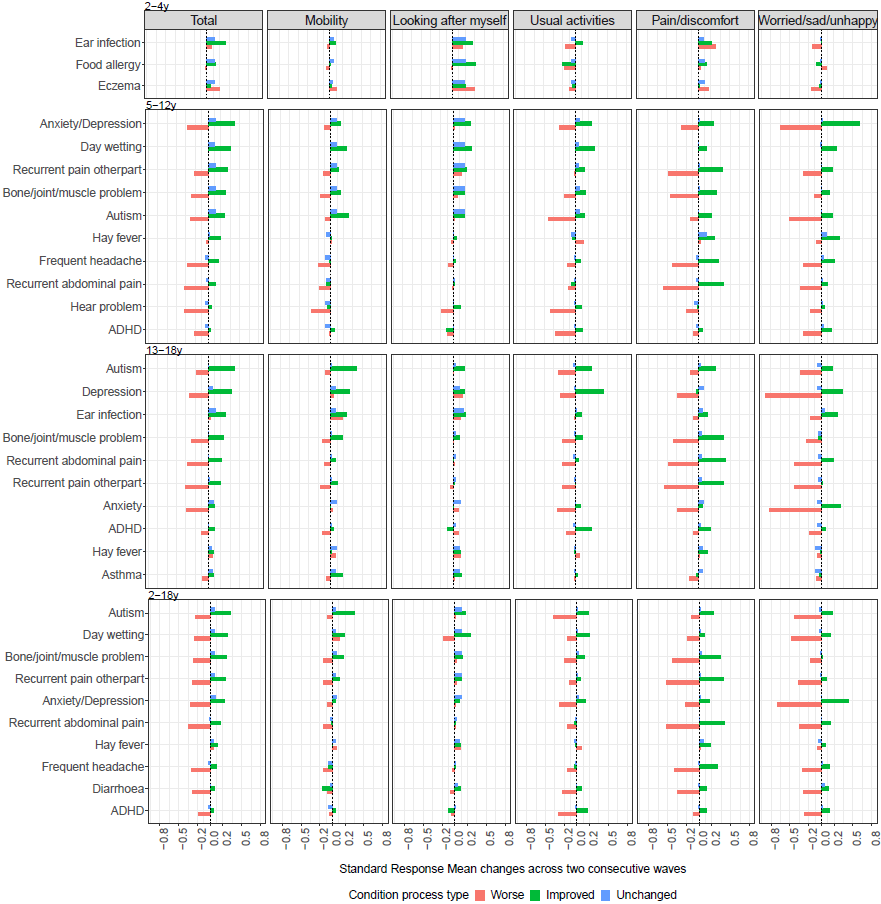
**Appendix 4 Figure 1** The total and dimension score changes (SRM) of inferred EQ-5D-Y over 2 years


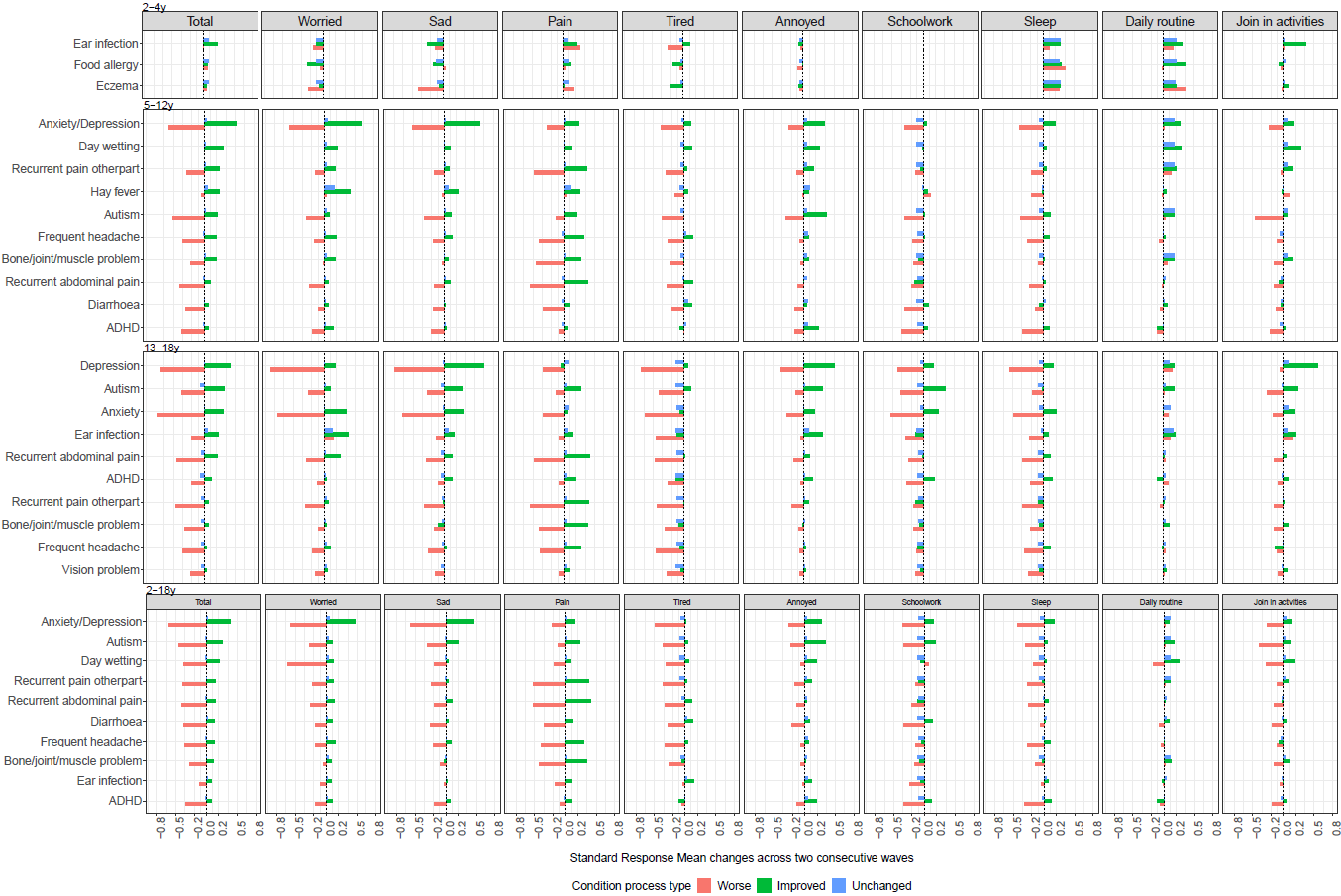
**Appendix 4 Figure 2** The total and dimension score changes (SRM) of inferred CHU9D over 2 years

# Appendix 5 – Sensitivity analysis: including a narrower set of items in dimensions

**Appendix 5 Table 1** Items included in dimensions in sensitivity analysis of EQ-5D-Y and CHU9D

| EQ-5D-Y dimension s1 | PedsQL items |
| --- | --- |
| Mobility_s | 1.1 Walking more than 100 meters  1.2 Running |
| Sad and Worried_s | 1.8 Having a low energy |
|  | 2.1. Feeling afraid or scared |
|  | 2.2 Feeling sad |
|  | 2.3 Feeling angry |
|  | 2.4 Having trouble sleeping |
|  | 2.5 Worrying about what will happen to him or her |
| Usual Activities_s1 | 1.3. Participating in sports activity or exercise |
|  | 1.6. Doing chores around the house |
|  | 3.4. Not being able to do things that other children his or her age can do |
|  | 3.5 Keeping up when playing with other children |
|  | 4.3 Keep up with school activities/ schoolwork |
| Usual Activities_s2 | 1.3. Participating in sports activity or exercise |
|  | 1.6. Doing chores around the house |
|  | 3.4. Not being able to do things that other children his or her age can do |
|  | 3.5. Keeping up when playing with other children |
|  | 4.3 Keep up with school activities/ schoolwork |
| Usual Activities_s3 | 1.3 Participating in sports activity or exercise |
|  | 1.6. Doing chores around the house |
|  | 3.4. Not being able to do things that other children his or her age can do |
|  | 3.5. Keeping up when playing with other children |
|  | 4.3 Keep up with school activities/ schoolwork |
|  |  |
| CHU9D dimension s1 | PedsQL items |
| Worried_s | 2.1 Feeling afraid or scared |
|  | 2.5 Worrying about what will happen to him or her |
| Join in activities_s (playing out with friends, doing sports, joining things) | 1.3 Participating in sports activity or exercise |
|  | 3.1 Getting along with other children |
|  | 3.5 Keeping up when playing with other children |

**Note:** The grey items were included in the main paper but were excluded in the sensitivity analyses.

Including a narrower set of items to calculate dimension scores generally does not change much of the total scores, although there is some difference in dimension scores.


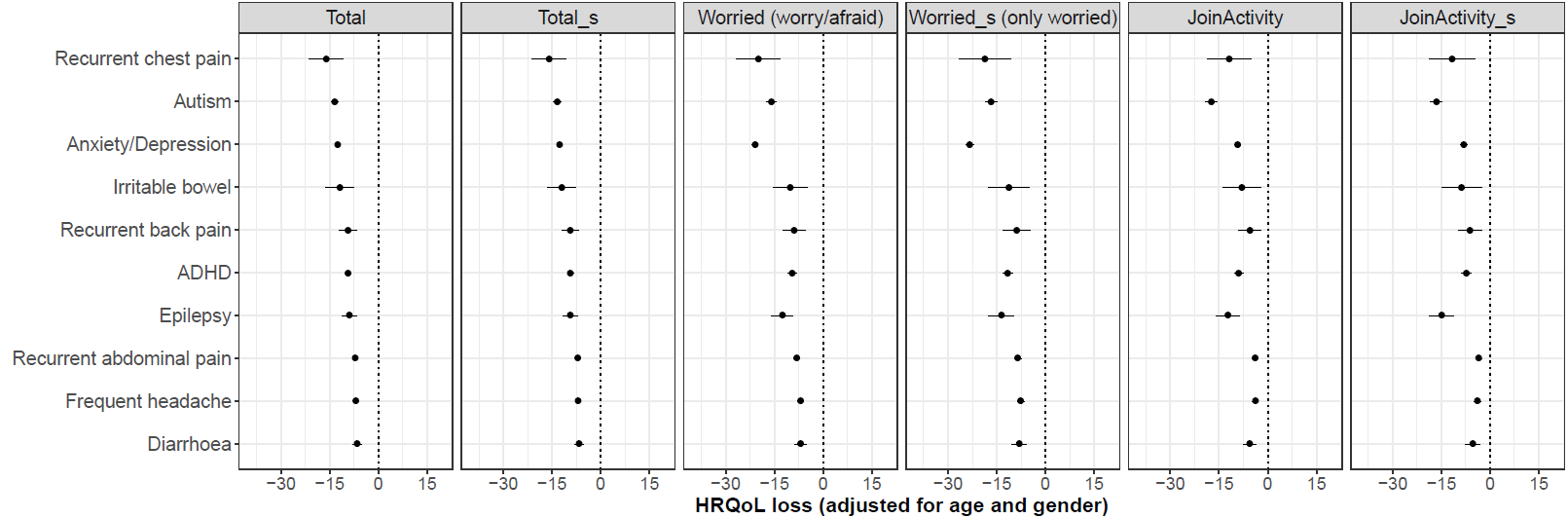

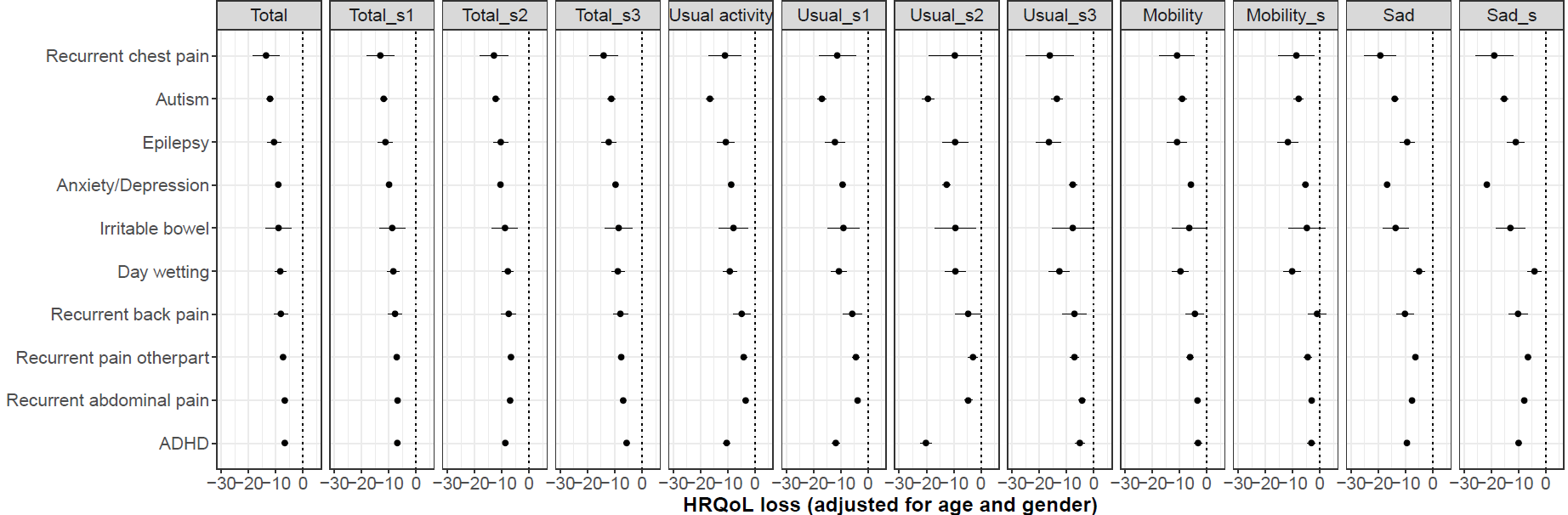
**Appendix 5 Figure 1** Compare HRQoL loss measured by inferred EQ-5D-Y when including different items in 2-18 years old (Total_s1 uses Mobility_s, Sad/worry_s and Usual_s1. Total_s2 and Total_s3 only differed by using Usual_s2 and Usual_s3)

**Appendix 5 Figure 2** Compare HRQoL loss measured by inferred CHU9D when including different items in 2-18 years old (‘Total_s’ incldued ‘Worried_s’ and ‘JoinActivity_s’’.)


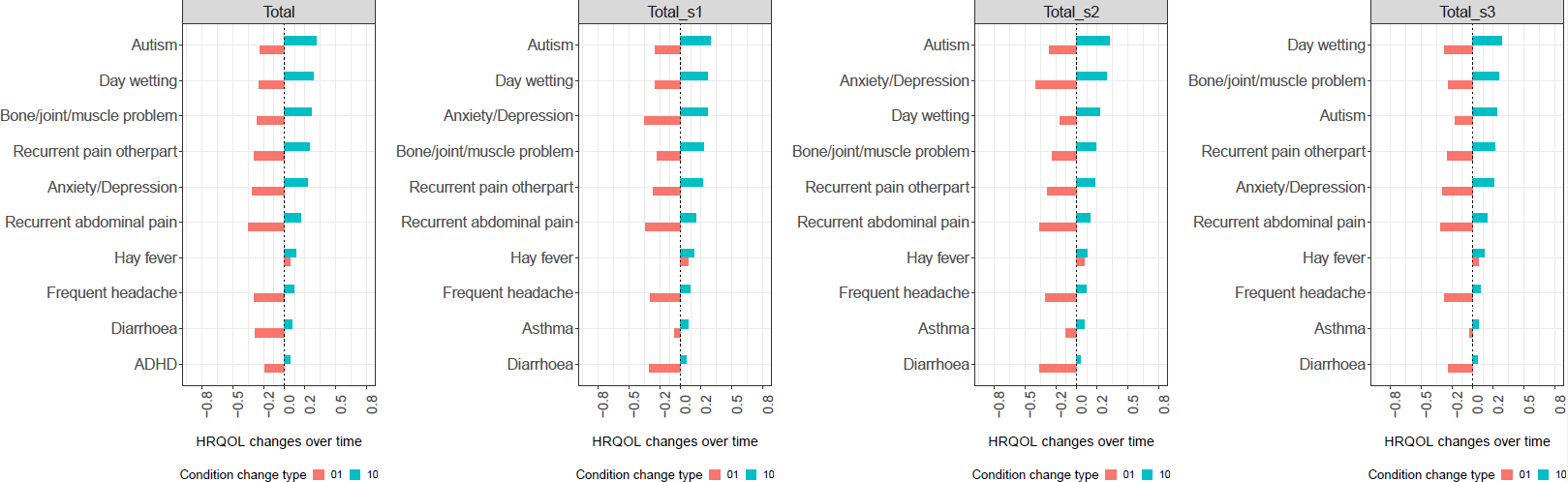
The top 10 conditions with largest HRQoL improvement are generally the same in different total scores, only the last condition differed. The rank of conditions differed a bit (as expected).

**Appendix 5 Figure 3** Compare top 10 conditions with the largest inferred EQ-5D-Y total score changes over time when including different items in dimensions in 2-18 years old (Total_s1 includes Usual_s1. Total_s2 includes Usual_s2. Total_s3 includes Usual_s3)

# Appendix 6 – Using real CHU9D data to estimate HRQoL impairment


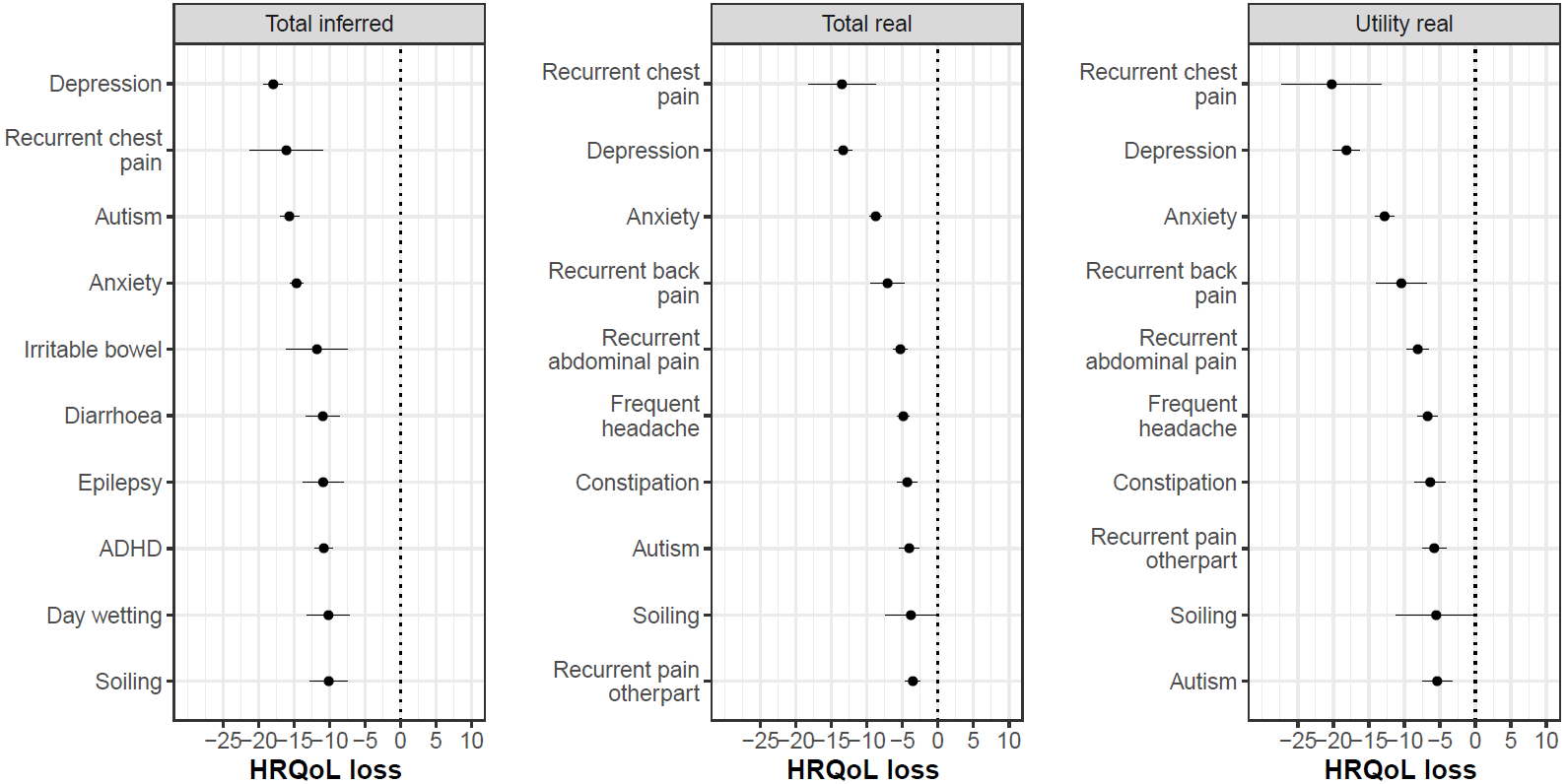


**Appendix 6 Figure 1** Comparing the top 10 conditions with largest HRQoL impairment associated with health conditions between inferred and real CHU9D


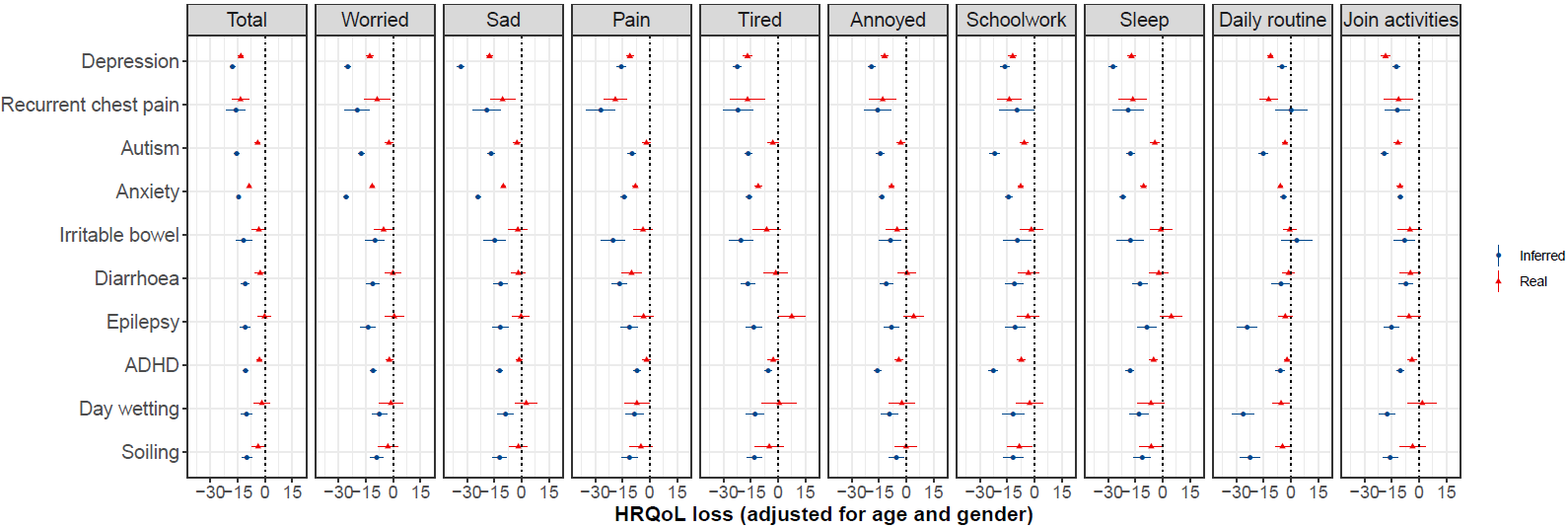


**Appendix 6 Figure 2** Compare HRQoL impairment between inferred and real CHU9D average total scores

**Notes:** CHU9D utility were calculated by Australian adolescent value set^5^. CHU9D utilities was transformed to 0-100 by multiply the original utility with 100 to have comparable regression coefficients with CHU9D like score. For *daily* *routine*, the inferred CHU9D had a simpler description than the real CHU9D. Real CHU9D had *Daily routine (eating, having a bath/ Shower, getting dressed),* while inferred CHU9D had *4.3.Taking a bath or shower by him or herself.*

# Reference

1. Schober P, Boer C, Schwarte LAJA, Analgesia. Correlation coefficients: appropriate use and interpretation. 2018; **126**(5): 1763-8.

2. Petersen KD, Ratcliffe J, Chen G, Serles D, Frosig CS, Olesen AV. The construct validity of the Child Health Utility 9D-DK instrument. *Health and quality of life outcomes* 2019; **17**(1): 187.

3. Mulhern B, Mukuria C, Barkham M, et al. Using generic preference-based measures in mental health: psychometric validity of the EQ-5D and SF-6D. *British Journal of Psychiatry* 2018; **205**(3): 236-43.

4. Burström K, Bartonek Å, Broström E, Sun S, Egmar A-C. EQ-5D-Y as a health-related quality of life measure in children and adolescents with functional disability in Sweden: testing feasibility and validity. *Acta Paediatrica* 2014; **103**(4): 426-35.

5. Ratcliffe J, Flynn T, Terlich F, Stevens K, Brazier J, Sawyer M. Developing Adolescent-Specific Health State Values for Economic Evaluation. *PharmacoEconomics* 2012; **30**(8): 713-27.
